# Recent Development and Clinical Application of Cancer Vaccine: Targeting Neoantigens

**DOI:** 10.1155/2018/4325874

**Published:** 2018-12-19

**Authors:** Ren-You Pan, Wen-Hung Chung, Mu-Tzu Chu, Shu-Jen Chen, Hua-Chien Chen, Lei Zheng, Shuen-Iu Hung

**Affiliations:** ^1^Department of Dermatology, Drug Hypersensitivity Clinical and Research Center, Chang Gung Memorial Hospital, Linkou, Taipei and Keelung, Taiwan; ^2^Chang Gung Immunology Consortium, Chang Gung Memorial Hospital and Chang Gung University, Taiwan; ^3^College of Medicine, Chang Gung University, Taoyuan, Taiwan; ^4^Whole-Genome Research Core Laboratory of Human Diseases, Chang Gung Memorial Hospital, Keelung, Taiwan; ^5^Department of Dermatology, Xiamen Chang Gung Hospital, China; ^6^Department and Institute of Pharmacology, School of Medicine, National Yang-Ming University, Taipei, Taiwan; ^7^ACT Genomics, Taipei, Taiwan; ^8^The Pancreatic Cancer Precision Medicine Center of Excellence Program, Johns Hopkins University School of Medicine, Baltimore, Maryland, USA

## Abstract

Recently, increasing data show that immunotherapy could be a powerful weapon against cancers. Comparing to the traditional surgery, chemotherapy or radiotherapy, immunotherapy more specifically targets cancer cells, giving rise to the opportunities to the patients to have higher response rates and better quality of life and even to cure the disease. Cancer vaccines could be designed to target tumor-associated antigens (TAAs), cancer germline antigens, virus-associated antigens, or tumor-specific antigens (TSAs), which are also called neoantigens. The cancer vaccines could be cell-based (e.g., dendritic cell vaccine provenge (sipuleucel-T) targeting prostatic acid phosphatase for metastatic prostate cancer), peptide/protein-based, or gene- (DNA/RNA) based, with the different kinds of adjuvants. Neoantigens are tumor-specific and could be presented by MHC molecules and recognized by T lymphocytes, serving the ideal immune targets to increase the therapeutic specificity and decrease the risk of nonspecific autoimmunity. By targeting the shared antigens and private epitopes, the cancer vaccine has potential to treat the disease. Accordingly, personalized neoantigen-based immunotherapies are emerging. In this article, we review the literature and evidence of the advantage and application of cancer vaccine. We summarize the recent clinical trials of neoantigen cancer vaccines which were designed according to the patients' personal mutanome. With the rapid development of personalized immunotherapy, it is believed that tumors could be efficiently controlled and become curable in the new era of precision medicine.

## 1. Introduction

Cancer cells have characteristics of genetic instabilities and accumulate somatic mutations rapidly (1–4). The genome sequencing of cancer cells revealed heterogeneity, and tens to hundreds to thousands of somatic mutations amassed in individual patients. The high intertumoral heterogeneity is evidenced by The Cancer Genome Atlas (TCGA) database, which stores the genomic data of thousands of tumor specimens [1–3]. There are various types of mutations, such as point mutations, insertion/deletions, gene amplification, and translocations in cancer cells. Some of them may lead to nonsynonymous somatic mutations altering the amino acid coding sequences and creating uncontrollable and abnormal proteins to promote cell proliferation. These aberrant peptide sequences could be seen by our immune system. Tumor-specific antigens (TSAs), called as neoantigens, are created by the genomic codon alternations, editing, usage, antigen processing, and presentation [4, 5]. Neoantigens could be presented by the major histocompatibility complex (MHC; also known as human leukocyte antigen (HLA) in humans) on the cell surface and recognized by the T lymphocytes. As neoantigens are tumor-specific and not expressed by normal cells [4, 5], they are ideal therapeutic targets and have great potential to maximize the therapeutic specificity, overcome the immune tolerance, and minimize the risk of autoimmunity. In this article, we review the literature of tumor antigens and cancer vaccines and also discuss the applications and values of this approach towards precision medicine.

## 2. Emerging Immunotherapies for Cancer Treatments

In recent years, immunotherapies rapidly develop and open a new era of cancer treatment. In 2011, the FDA first approved an immune checkpoint inhibitor (ICI), ipilimumab, a CTLA-4 blockage, which prolonged the overall survival rate of patients with metastatic melanoma [6, 7]. Following this line, there are increasing ICI, such as anti-PD1 and anti-PD-L1 antibodies, proven to be effective and durable therapies in subsets of patients with a variety of tumor types: metastatic melanoma, nonsmall cell lung cancer (NSCLC), prostate cancer, renal cell carcinoma, and so on [8]. The response rates of ICI, however, are correlated with the mutation load of tumors of individuals and the presence of microsatellite instability (MSI) or DNA repair enzyme deficiency [9–11]. Nevertheless, the use of ICI carries a risk to develop irAE (immune-related adverse events), which occur via nonspecific activation of the patient's immune system, leading to serious and even fatal adverse reactions [12, 13]. More efforts are needed to improve the response rates and tumor antigen specificity of ICI and to decrease the incidence of irAE. More recently, the first chimeric antigen receptor- (CAR-) T cell immunotherapy, anti-CD19 CAR-T for B cell lymphoma, was approved by the FDA in Aug 2017 [14, 15]. After that, there are increasing clinical trials using CAR-T therapy to treat cancers [16, 17]. CAR-T cells target the tumor-associated antigens (TAAs), such as CD19 on B cell malignancies [18, 19] and ERBB2 on breast cancers [20], which are also expressed on the normal cells. CAR-T therapy has the on-target but off-tumor side effect. Although CAR-T therapies have shown considerable promise in some acute lymphoid leukemia [18, 19], it is still a big challenge to treat solid cancers with CAR-T cells due to the lack of suitable TAAs. The reported overall objective response rates (ORR) of CAR-T therapy for solid tumors are still low [21, 22].

Targeting tumor-specific antigens (TSAs) has been considered an important therapeutic approach. As TSAs are exempt from central tolerance [23], these neoantigens could be presented by HLA and recognized by T lymphocytes of the immune system. Effective antitumor immunity in humans has been associated with the presence of T cells recognizing cancer neoantigens. The studies of adoptive cell transfer (ACT) of autologous tumor-infiltrating lymphocytes (TILs) revealed that neoantigen-specific T cells are crucial for clinical responses [24–27]. The isolated T cell clones or T cell receptor- (TCR-) engineered T lymphocytes demonstrated the epitope patterns of neoantigens recognized by T cells [28–30]. There are increasing neoantigen-based cancer vaccines designed to target the unique immunogenic mutations arising in each patient's tumor [31]. Recently, two groups showed glimmers of the success of personalized cancer vaccines [32, 33]. Both the personalized RNA mutanome vaccines and peptide-based vaccines induced poly-specific therapeutic immunity against cancer [32, 33]. These neoantigen cancer vaccines demonstrated to be relatively safe, feasible, and capable of eliciting strong T cell responses to neoepitopes in patients with melanoma [32, 33]. Treatments tailored to a person's individual cancer mutations cause the strong immune response to attack tumors.

## 3. Tumor Antigens for Immunotherapy

Regarding the targets of immunotherapy, there are different types of tumor antigens, including tumor-associated antigens (TAAs), cancer germline antigens (CGAs), virus-associated antigens, and tumor-specific antigens (TSAs) (Table 1).

Tumor-associated antigens (TAAs) are present in normal cells with low levels of expression but overexpressed on tumor cells in different patients. There are different kinds of TAAs, e.g., carcinoembryonic antigen (CEA) for GI cancer and PAP for prostate cancer. Using the universal antigens, various cancer vaccines have been designed for patients with tumor expressing the specific TAA. For example, stimuvax (BLP25 liposome vaccine) targeting MUC1 for NSCLC is in the phase III trial [34].

However, most attempts targeting TAAs in the cancer vaccination have met with limited success, as TAAs are normal host proteins and therefore subject to both central and peripheral tolerance mechanisms [35]. Due to the positive and negative selection, the high-affinity TCRs for TAAs are preferentially depleted, and the affinities of the remaining TCRs for TAAs are lower than that of the TCRs for foreign antigens [36, 37]. In addition, targeting TAAs may cause autoimmune toxicities, such as colitis, severe hepatitis, renal impairment, rapid respiratory failure, and even death [38]. For example, targeting “carbonic anhydrase 9” caused severe liver toxicity, as this TAA is expressed in bile duct epithelial cells. Nevertheless, using TAAs as immunotherapy targets still has its clinical value. CAR-T therapy targeting CD19 in patients with acute lymphoblastic leukemia (ALL) showed complete remission in high proportion of patients, though life-long administration of IVIG is needed for the patients [14, 15].

Cancer germline antigens (CGAs), also called cancer/testis antigens (CTAs), are present in reproductive tissues, such as testes, fetal ovaries, and trophoblasts, but have limited expression on other normal tissues in adults and are generally not present on normal reproductive cells (Table 1) [35, 39]. CGAs, such as melanoma-associated antigen 3 (MAGE-A3) and NY-ESO-1 antigen, are selectively expressed by various cancers [40, 41]. However, attempts to target CGAs have met hurdles. For example, targeting MAGE-A3 resulted in severe neurological toxicity and death [42].

Some cancers have been associated with virus infection, and the viral-encoded antigens comprising the viral open reading frames are present in the tumors only but not the normal cells (Table 1). The viral oncogenes encode oncoproteins and cause cell transformation and tumorigenesis, such as Merkel cell polyomavirus- (MCPyV-) associated Merkel cell carcinoma (MCC) and human papillomavirus- (HPV-) associated cervical cancer or oropharyngeal cancer [43–45]. Targeting virus-associated antigens has been considered to be one of the effective methods for treating cancers [46–48]. Nevertheless, some virus-associated antigens showed ability to escape from the immune detection of the host [49, 50].

Tumor-specific antigens (TSAs; neoantigens) arise from nonsynonymous mutations and other genetic alterations in cancer cells (Table 1). Neoantigens are mutated peptides been presented by HLA on the cell surface and subsequently recognized by the immune system. TSAs are theoretically more attractive therapeutic targets because they are different from the germline and seen as nonself by the immune system. Because normal cells do not express TSAs, neoantigen-specific immune reactions are not subject to central and peripheral tolerance. In addition, targeting TSAs should be less likely to induce autoimmunity. As a result, neoantigens appear to represent the ideal targets for therapeutic cancer vaccine and T cell-based cancer immunotherapy. Several neoantigens have been identified from different types of cancers, including melanoma, lung cancer, hepatoma, and renal cancers [51, 52].

## 4. Categories of Cancer Vaccines

With the development of technologies of next-generation sequencing (NGS), it becomes apparent that human cancers are very complex, bearing thousands of mutations. By the application of platforms of immune repertoire, increasing evidence reveals that some of the tumor antigens could be recognized by the immune repertoire. Now, there are different prediction algorithms and software for the epitope mapping and MHC/neoantigen binding [5, 53]. Different kinds of cancer vaccines could be designed to target diverse tumor antigens, including shared antigens or private epitopes.

**Figure 1:**
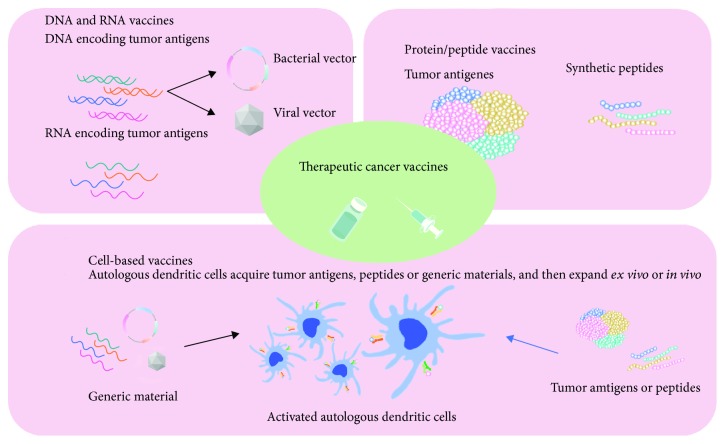
Schematic representation of different types of therapeutic cancer vaccines, which could be designed according to the forms of cells, proteins/peptides, and genes.

There are three broad types of cancer vaccines, designed in the forms of cells, proteins/peptides, and genes (Figure 1). Regarding cell-based cancer vaccines, there are (1) autologous or allogeneic whole tumor cell vaccine and (2) autologous dendritic cells (DC), pulsed or transfected with tumor antigens in different forms, such as tumor lysates, purified proteins, peptides, DNA, or RNA [54]. When using the whole tumor cells as the antigens, the cells could be inactivated by heat, chemicals, or radiation. There are different kinds of cancer vaccines using whole tumor cells, e.g., OncoVAX (Vaccinogen) for colon cancer, Reniale (LipoNova) for renal cancer, and GVAX for prostate cancer [55–57]. The autologous or allogenic whole tumor cells can be genetically modified to produce immune molecules, e.g., Lucanix (belagenpumatucel-L from NovaRx) for NSCLC [58]. The phase III study of Lucanix (belagenpumatucel-L from NovaRx), however, failed to meet the endpoint in NSCLC [58]. Since the main disadvantage of whole tumor cell-cancer vaccine is nonspecificity, targeting TAA as the component of the cell-based vaccine may improve the anticancer effect. For example, the dendritic cell vaccine, provenge (sipuleucel-T), targeting PAP for metastatic castration-resistant prostate cancer, was the first FDA-approved cell-based cancer vaccine in 2010 [59]. Nevertheless, cell-based vaccines also have the limitations of the high-cost, time-consuming, and large-scale manufacturing production for individual patients [60, 61].

Protein/peptide-based vaccines could be composed of TAAs, CGAs, virus-associated antigens, or TSAs, with different adjuvants. The synthetic peptide vaccines are usually composed of 20–30 amino acids targeting the specific epitopes of tumor antigens. Furthermore, the tumor antigens could be modified to fuse or mix with cytokines, antibodies, or immunogenic peptides in the protein/peptide-based cancer vaccines, e.g., Oncophage for kidney cancer, melanoma, and brain cancer and Stimuvax (BLP25 liposome vaccine) targeting MUC1 for NSCLC and breast cancer [34, 62, 63]. Peptide vaccines have several advantages, such as easy synthesis with low cost, increased stability, and relative safety. Peptide vaccines have been generally demonstrated in numerous preclinical and clinical studies. However, there are obstacles of peptide vaccines needed to be overcome, which include the limitation of well-known peptide epitopes as vaccine candidates, immune evasion, weak immunogenicity of tumor antigens, and high cost for cGMP manufacturing and production of a fully personalized cancer vaccine [64–66].

Gene-based cancer vaccines apply DNA (as plasmids) or RNA (as mRNA), which could be taken up by antigen-presenting cells (APC) and translated into peptides or proteins as cancer-specific antigens to stimulate the immune response. There are different kinds of DNA cancer vaccines, such as mammaglobin-A for breast cancer, PAP for prostate cancer, gp100 and gp75 DNA for melanoma, and VXM01 for pancreatic cancer [59, 67–70]. The major obstacle of gene-based vaccination is the DNA/RNA delivery method and uptake efficiency, consequently limiting the antigen transcription and presentation by APC [71]. Although electroporation or viral vectors showed higher efficiency to deliver the DNA or RNA into cells, both methods are difficult to be applied in clinical practice [72–75]. For example, the clinically approved devices for electroporation are available; however, patients' compliance has limited the use [73]. Regarding the virus-mediated delivery, it should be carefully considered for the potential side effects related to the administration of live virus together with the decreased efficiency of the presence of antiviral neutralizing antibodies in patients [72].

## 5. Preclinical and Clinical Trials Applying Neoantigen-Based Cancer Vaccines

**Figure 2:**
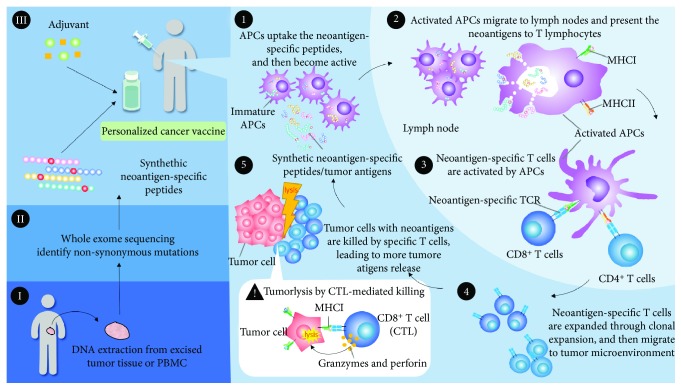
The designing strategies and immunology of personalized neoantigen cancer vaccine. (I–III) The tumor neoantigens of an individual are identified using the whole exome sequencing, and the personalized neoantigen cancer vaccine is introduced. (1) The APCs uptake the neoantigen peptides in the vaccination sites and then migrate to the lymph nodes. (2) The activated APCs present the neoantigens by MHC class I or MHC class II molecules to T cells. (3) The neoantigen-specific TCR recognizes the specific neoantigen presented by the MHC molecules of APCs. (4) The neoantigen-specific CD4^+^ helper or CD8^+^ cytotoxic T cells are activated and clonally expanded and then migrate to the tumor microenvironment. (5) The tumor cells are killed directly by neoantigen-specific CD8^+^ cytotoxic T cells, leading to the release of more of tumor neoantigens. APC: antigen-presenting cell; MHC: major histocompatibility complex; TCR: T cell receptor.

The number of somatic mutations ranges from a few dozens to several tens of thousands in an individual tumor. With the development of NGS technologies, the highly heterogeneous neoantigens of tumor cells could be characterized. The cancer vaccine is a relatively safe and effective therapy compared to other methods of cancer treatments. To generate the personalized cancer vaccine, the somatic mutations of cancer cells could be identified by the whole exome sequencing via the comparison of the genomic DNA data of excised tumor tissue and peripheral blood mononuclear cells (PBMC) of an individual. According to the profile of detected tumor mutations, the personalized cancer vaccine could be designed to target the specific epitopes of neoantigens against cancers. The personalized cancer vaccine may consist of the synthetic peptides or genes encoding the shared tumor antigens, or private neoantigens, with the presence of adjuvants such as poly-ICLC, GM-CSF, and BCG (Figure 2). Personalized cancer may be used with the combination of other therapeutics, e.g., ICI, chemotherapy, or radiation therapy.

Based on the theory of tumor-immune cell interaction, the personalized cancer vaccination works to activate the immune system and kill cancers (Figure 2) [76]. First, the neoantigens from the cancer vaccine or died cancer cells are captured by APCs. Next, the activated APCs migrate to the lymph nodes and the MHC molecules present the neoantigens to T lymphocytes. The specific TCR recognizes the neoantigens, resulting in the priming and activation of T cell immunity. Neoantigen-specific T cells are then expanded, traffic and infiltrate to the tumor microenvironment. These expanded T cells specifically bind to the neoantigens of cancer cells via the interaction of the TCR/neoantigen/MHC complex. The CD4-positive T cells augment the immune response against cancers, and CD8-positive cytotoxic T lymphocytes (CTL) directly kill the cancer cells through the degranulation of granzyme, granulysin, or perforin. The lysed tumor cells release more neoantigens, which elicit the adaptive immune memory response and lead to the expansion of molecularly heterogeneous T cells against cancers (Figure 2).

In the preclinical studies of the tumor vaccination using a mouse model, Castle et al. explored the mutanome and identified candidate mutated epitopes by whole exome sequencing of the B16F10 murine melanoma (46). Fifty selected mutated gene coding peptides were vaccinated to mice, and 11 of 50 peptides demonstrated immunogenicity and induced immune responses (46). The mutated Kif18b (K739 N) was the dominant mutated antigen, and mice immunized with mutated Kif18b peptide showed decreased tumor progression and improved survival [77]. Yadav et al. predicted the immunogenic tumor mutations by combining mass spectrometry and exome sequencing (47). MC-38 tumor-bearing mice, which were injected with the mutated peptide vaccine (Adpgk, Reps1, and Dpagt1), showed the suppression of tumor growth [78]. Castle et al. developed a synthetic RNA pentatope vaccine (36). Each pentatope contained five 27-mer minigenes, including the mutated amino acids in the center, and each pentatope was fused to another by 10-mer glycine-serine linker (36). The CT26 tumor-bearing mice were vaccinated with the RNA pentatope, and slow disease progression and improved survival were observed (36). This study suggests that mutant MHC class II epitopes are more immunogenic and drive therapeutic immune response to cancer than that of class I epitopes.

There are clinical trials evaluating the safety and efficacy of personalized cancer vaccines. Some of the clinical trials have shown encouraging results. For example, Carreno et al. identified somatic mutations in tumors from 3 patients with melanoma by whole exome sequencing (48). The authors used an HLA binding prediction algorithm to initially filter the candidate *HLA-A*^∗^*02 : 01* epitopes containing residues arising from mutations and then evaluated the MHC-epitope binding using competitive assays. The three patients received autologous dendritic cells pulsed with the top 7 neoantigen peptides, which showed higher binding affinity to the *HLA-A*^∗^*02 : 01*. They found that dendritic cell neoantigen vaccine increased the diversity of melanoma neoantigen-specific T cells (48). These neoantigens could be endogenously processed and presented to T cells, and the T lymphocytes elicited by vaccination could recognize the target cells transfected with the corresponding tandem minigene constructs [79]. Recently, Ott et al. enrolled 6 patients with melanoma and identified the tumor-specific mutations by NGS [33]. To make the personalized peptide vaccines, the authors predicted the neoantigens which could bind to the individual MHC proteins by algorithms. Each patient was vaccinated using the synthetic long peptides representing up to 20 predicted personal tumor neoantigens. The vaccination induced polyfunctional CD4^+^ and CD8^+^ T cells targeting 58 (60%) and 15 (16%) of the 97 unique neoantigens used across patients [33]. These T cells could discriminate mutated and wild-type antigens, and some of them could directly recognize autologous tumor [33]. Four of 6 patients had no recurrence at 25 months after vaccination, and the other two patients with recurrent disease were subsequently treated with anti-PD-1 therapy and experienced complete tumor regression [33]. In addition, Sahin et al. showed that personalized RNA mutanome vaccines elicited poly-specific therapeutic immunity against melanoma [32]. This study applied a process comprising the comprehensive identification of individual mutations, computational prediction of neoantigens with high binding affinity to MHC proteins, and designing and manufacturing of an RNA-based vaccine unique for each patient [32]. All patients developed T cell response against multiple vaccine neoantigens [32]. The cumulative rate of metastatic events was significantly reduced after the injection of vaccine, resulting in a sustained progression-free survival [32]. Two of 5 patients with metastatic disease had vaccine-related objective response, and a patient developed a complete response to vaccination in combination with PD-1 blockade therapy [32]. These promising results demonstrate that personalized neoantigen cancer vaccine opens a new path to cure the disease.

## 6. Conclusion and Future Perspectives

Cancer vaccine composed of unique tumor antigens specifically forces the immune system to recognize the malignancies, which could be used alone or in combination with other therapies. Among the different kinds of tumor antigens, neoantigens are ideal therapeutic targets for the design of cancer vaccine as they are tumor-specific and have the lowest risks of autoimmunity. The neoantigen-based cancer vaccines showed the induction of de novo T cell clones that detected multiple individual-specific neoantigens and recognized endogenously processed antigens and autologous tumor cells [32, 33]. When the encouraging results of personalized cancer vaccines are accumulating, there are some obstacles needing to overcome. Some cancers are “cold tumors,” e.g., pancreatic cancers and colorectal cancers, showing low response rates to immunotherapies. How to use personalized cancer vaccines to increase the reactive T cells in the microenvironment and combine with other therapies to have synergy effects on “cold tumors” needs further investigation. Another concern is the heterogeneity of tumor and immune escape. In an individual patient, the same types of neoantigens may be expressed on some, but not all tumor cells, which may cause cancers to escape from immunotherapy. One potential approach to solve this problem is to target multiple neoantigens of a diversity of malignant clones per patient, as demonstrated in the previous studies [32, 33]. Therefore, all tumor cells could be destroyed at the same time of the treatment course, and the cancer vaccine minimizes the chance of tumor escape by the loss of antigens [32, 33]. Lastly, the pharmacoeconomics is also an important issue for implementing personalized neoantigen cancer vaccine into clinic practice. The individualized vaccine is still expensive due to the cost for genome sequencing and manufacturing of small and personalized-specific GMP drug product batches. However, the expense for personalized cancer vaccine may reduce following the development of improved methods for predicting antigen presentation, the process of commercialization, full automation, and optimization of manufacturing processes. After having more understandings of the cancer immunology, the cancer vaccine may be designed to target the driver mutations or shared antigens of different tumor types or individuals, to increase the therapeutic efficiency, and to reduce the expense of manufacturing [80]. In conclusion, there are increasing evidence demonstrating the feasibility, safety, and immunogenicity of the personalized cancer vaccine in the treatment of cancer patients. The personalized cancer vaccine could work alone or in combination with other therapies to enhance the strength and persistence of antitumor effects, increase the survival rates and quality of life, and ultimately improve the efficacy of cancer treatments in the patients. It is anticipated that personalized cancer vaccine will make precision medicine to be available and affordable for most of patient population in the near future.

## Figures and Tables

**Table 1:** Categories of tumor antigens.

Different antigen types, descriptions, and examples
Tumor-associated antigens (TAAs)
Low levels of expression on normal host cells
Disproportionately expressed on tumor cells
Often result from genetic amplification or posttranslational modifications
Example: CD19 on B cell malignancies
Cancer germline antigens (CGAs)/cancer testis antigens (CTA)
Absent on the normal adult cells, except in reproductive tissues such as testes, fetal ovaries, and trophoblast
Selectively expressed by various tumor types by epigenetic dysregulation
Example: NY-ESO-1 in various tumors
Virus-associated antigens
Arise in cancer cells from oncogenic viral proteins
Viral oncoproteins integrate into host cell genome, causing cell transformation and tumorigenesis
Carried by virally associated malignancies
Example: HPV E6/E7 oncoproteins
Tumor-specific antigens (TSAs)/neoantigens
Arise in cancer cells from nonsynonymous somatic mutations that result in the formation of new peptide sequences during tumorigenesis
Completely absent from normal host cells
Example: individual KRAS G12D somatic mutation

## Abbreviations

ACT: Adoptive cell transfer
APC: Antigen-presenting cells
CAR-T therapy: Chimeric antigen receptors-T cell therapy
CGAs: Cancer germline antigens
CTL: Cytotoxic T lymphocyte
HLA: Human leukocyte antigen
ICI: Immune checkpoint inhibitors
irAE: Immune-related adverse events
MHC: Major histocompatibility complex
NSCLC: Nonsmall cell lung cancer
NGS: Next-generation sequencing
TAA: Tumor-associated antigen
TCR: T cell receptor
TIL: Tumor-infiltrating lymphocytes
TSA: Tumor-specific antigen.
